# An improved CRISPR-Cas9 protein-based method for knocking out insect Sf9 cell genes

**DOI:** 10.1007/s00253-026-13722-3

**Published:** 2026-01-26

**Authors:** Miguel Graça, Nikolaus Virgolini, Ricardo Correia, Jose Escandell, António Roldão

**Affiliations:** 1 iBET, Instituto de Biologia Experimental E Tecnológica, Apartado 12, Oeiras, 2780-901 Portugal; 2 Instituto de Tecnologia Química E Biológica António Xavier, Universidade Nova de Lisboa, Av. da República, Oeiras, 2780-157 Portugal

**Keywords:** *Spodoptera frugiperda*, Baculovirus expression vector system, Cell line development, CRISPR-Cas9, Apoptosis, Advanced therapy medicinal products

## Abstract

**Abstract:**

Insect cells are one of the uprising expression systems in the biopharmaceutical industry to produce vaccines and gene therapy vectors, but cell line development has been limited by the lack of established genetic engineering tools and genomic characterization. CRISPR-Cas9 has arisen as a powerful tool for gene editing but has seen little application in insect cells. In this work, a gene editing pipeline for the delivery of a ribonucleoprotein (RNP) complex comprised of a guide RNA and the enzyme Cas9 to insect Sf9 cells was implemented and then applied to knockout caspase initiator Sf-Dronc, aiming at alleviating cell apoptosis during an infection process. The resulting engineered cell lines were characterized as per their phenotype and production of three different product modalities. Utilizing the established workflow, a knockout rate of 68% was achieved with the implemented protocol (vs. the 12% presumed efficiency of a previously reported system) when targeting the *fdl* gene. When applied to *Sf-Dronc*, mutants containing deletions in several alleles of the host genome were identified and confirmed by next-generation sequencing. Generated clones exhibited higher apoptosis resistance and delayed onset of cell viability drop following infection with baculovirus. While *Sf-Dronc* deletion was shown to have negligible impact on the production of rAAV and PfRipr5, production of iVLPS showed an > twofold increase over wild-type Sf9. Overall, this study showcases the successful implementation of an efficient CRISPR-Cas9 pipeline, further leveraging the usage of genetic engineering in insect Sf9 cells towards the development of enhanced cell hosts for biopharmaceutical production.

**Key points:**

• *Implementation of an efficient CRISPR-Cas9 RNP complex delivery strategy to insect cells.*

• *Establishment of the genome editing pipeline demonstrated through Sf-Dronc knockout, resulting in increased apoptosis resistance and delayed loss of viability upon baculovirus infection.*

• *Sf-Dronc deletion led to over a twofold increase in the production of influenza VLPs compared to wild-type Sf9 cells.*

**Supplementary information:**

The online version contains supplementary material available at 10.1007/s00253-026-13722-3.

## Introduction

The insect cell–baculovirus expression vector system (IC-BEVS) has become a viable alternative expression system for industrial-scale production of therapeutic proteins, vaccines, and gene therapy vectors. In order to better leverage this system into industry and increase productivity, efforts have been made in bioprocess (Fernandes et al. 2020, 2022; Correia et al. 2020, 2022) and baculovirus engineering (Hong et al. 2022). However, improvements in titres through host cell engineering have been limited (Hong et al. 2022). Cell line development in insect cells has focused on improving N-glycan to better mimic humanized proteins, i.e. by knockout of the *fdl gene* encoding for the enzyme responsible for the removal of the N-terminal acetylglucosamine (Mabashi-Asazuma and Jarvis 2017). Another common strategy aims at delaying apoptosis during the infection process by knocking out or knocking down caspases involved in the apoptotic cascade or expressing anti-apoptotic genes (Lai et al. 2012; Steele et al. 2017; Zhang et al. 2018; de Malmanche et al. 2022), with the objective of increasing cell life span throughout the production phase and consequently improving titres. Unfortunately, this strategy has achieved limited success, largely due to the redundancy of multiple caspases existing in the *Spodoptera frugiperda* (Sf9) cell line and the inherent capacity of the baculovirus to inhibit host gene expression (Mabashi-Asazuma and Jarvis 2017; Zhang et al. 2018, 2021b; de Malmanche et al. 2022).

A recent milestone in genome editing was the discovery of CRISPR-Cas9, a RNA-guided Cas9 nuclease technology adapted from the microbial clustered regularly interspaced short palindromic repeats (CRISPR) adaptive immune system (Jinek et al. 2012). CRISPR-Cas9 has been demonstrated to make precise cuts to target genome sequences, creating indels and subsequently frame-shift mutations that inhibit gene expression (Adli 2018). As such, CRISPR-Cas9 gene editing has great promise in improving industry relevant cell lines (Grav et al. 2017). This technology has been applied to modify insect Sf9 cells (Mabashi-Asazuma and Jarvis 2017), e.g. establishing caspase-1 knockout clones (de Malmanche et al. 2022), highlighting an important milestone for that cell line and for the IC-BEVS. Despite this success, the reported plasmid-based approach has been associated with limitations, such as low editing efficiencies and unwanted plasmid DNA insertions, among others (Mabashi-Asazuma and Jarvis 2017; Zhang et al. 2021a). This low efficiency is partially attributed to the U6 promoter, which is less effective at driving gRNA expression in Sf9 cells, resulting in a time-consuming process (Mabashi-Asazuma and Jarvis 2017). As such, newer CRISPR-Cas9 delivery approaches in insect Sf9 cells are needed to accelerate the usage of genetic engineering in enhancing production processes.

Direct delivery of single-guide RNA (sgRNA) and Cas9 as a ribonucleoprotein (RNP) complex has emerged as a powerful and widely adopted alternative in CRISPR/Cas9 genome editing in several organisms. Delivering the system in RNP format addresses many of the drawbacks associated with plasmid-based methods, including reduced off-target effects, a lower risk of integration, and improved editing efficiencies (Zhang et al. 2021a; Du et al. 2023).

Taking advantage of this delivery format, the goal of this study was the implementation of a CRISPR/Cas9 RNP complex delivery strategy in Sf9 insect cells. Several transfection methods were tested to efficiently deliver RNP complexes to the cells while maintaining high viability post-transfection. Moreover, we also assessed the applicability of this protocol by knocking out the initiator caspase gene *Sf-Dronc*, recently identified to be differentially expressed in infection processes and responsible for signalling for the activation of effector caspases such as Sf-caspase-1 (Huang et al. 2013; Virgolini et al. 2023). Established clones were evaluated for improved apoptosis resistance as well as the production of three different biologics: a recombinant adeno-associated virus (rAAV), a virus-like particle displaying the influenza M1 protein (hereby named iVLPs), and a recombinant antigen against malaria (named PfRipr5).

## Materials and methods

### Cell lines and culture media

Sf9 cells (derived from *Spodoptera frugiperda*, Invitrogen, Cat#: 11496-015) and *Sf-Dronc* knockout clones were sub-cultured in serum-free Sf900-II™ SFM medium (Thermo Fisher Scientific) in shake flasks (Corning) using 10% (v/v) working volume, maintained at 27 °C and 100 rpm shaking in an Inova 44R shaking incubator (orbital motion diameter of 2.54 cm, Eppendorf). Cells were sub-cultured at 0.6–1 × 10^6^ cell.mL^−1^, when reaching a cell concentration of 2–4 × 10^6^ cell.mL^−1^. Cells maintained in culture plates (Corning) were sub-cultured when reaching confluency, using manual detachment and twofold dilution in fresh culture media. Culture plates were maintained in a static incubator at 27 °C.

### CRISPR-Cas9 knockout strategy

#### Ribonucleoprotein complex delivery

Single guide RNA (sgRNA) and Cas9 were purchased from IDT (Alt-R® CRISPR-Cas9 sgRNA and Alt-RTM S.p.Cas9 V3 products, respectively) and designed using IDT software or obtained from literature (Table S1). *Sf-Dronc* reference sequence (LOC118266887) was obtained from NCBI’s *S. frugiperda* Annotation Release 101 (RefSeq assembly accession: GCF_011064685.1) (Xiao et al. 2020). Ribonucleoprotein (RNP) complexes were formed by combining sgRNA with Cas9 according to the respective delivery method (lipofection or electroporation).

For lipofection, RNP complexes were formed during a 10-min incubation at room temperature using 15.5 pmol Cas9 and 30 pmol sgRNA in 250 µL Opti-MEM™ (Thermo Fisher Scientific). RNP vesicle formation was achieved by the addition of equal volumes of 6% (v/v) lipofection reagent (RNAiMax™, TransIT™, and Cellfectin™—all Thermo Fisher Scientific) in Opti-MEM™ and incubation at room temperature for 10 min. Finally, 0.6 × 10^6^ cell.mL^−1^ were transfected by adding 10% (v/v) delivery solution to each well.

For electroporation, 19.2 pmol Cas9 and 36 pmol sgRNA were complexed in 30 µL SF Nucleofector™ solution containing 18.2% (v/v) Nucleofector™ Supplement using the SF Cell Line 4D-Nucleofector™ X Kit (Lonza). After 15 min incubation at room temperature, 0.5 × 10^6^ cells were pelleted by centrifugation and resuspended in 20 µL nucleofection solution. Nucleofection was achieved in a 4D Nucleofector (Lonza) using a Nucleocuvette™ strip and the CM104 program. Immediately after nucleofection, 100 µL of fresh culture medium was added, and the cells was transferred to a culture plate.

#### Evaluating editing efficiency

To evaluate CRISPR-Cas9 editing efficiency, DNA was extracted with the DNeasy® Blood and Tissue Kit (Qiagen) according to the manufacturer’s instructions. Next, purified DNA was quantified using NanoDrop™ (Thermo Fisher Scientific), and 150 ng was subjected to subsequent target gene amplification using specific primers (primer pair 1 for *Sf-Dronc*, Table S1) at a concentration of 0.5 µM (Table S1) and PlatinumTM SuperFi II Green PCR Master Mix (Invitrogen) according to the manufacturer’s instructions. PCR conditions are shown in Table S2. Sequence amplification was confirmed by gel electrophoresis using a 1% agarose gel for 1 h at 100 V.

CRISPR-Cas9 editing efficiency was evaluated by using the EnGen® mutation detection kit (New England Biolabs) as instructed by the manufacturer. Briefly, the method involves PCR amplification of the target locus of edited cell DNA and then denaturing and re-annealing the PCR products. Heteroduplex DNA formed by annealed edited and nonedited amplimers is cleaved by T7 endonuclease. Briefly, 150 ng of target gene amplification product was digested using T7 endonuclease, and afterwards, reaction products were fractionated by polyacrylamide gel electrophoresis (PAGE) using a 4–20% gradient gel (TBE) (Invitrogen) for 1 h at 120 V in 0.5 × TBE buffer (Novex). Bands were then revealed by UV after staining with a 0.005% (v/v) aqueous RedSafe solution and subsequent washing steps using distilled water to reduce background signal. Editing efficiency was evaluated according to

$${\% efficiency}=100 \times [ 1-{\left(1-\text{fraction cleaved}\right)}^\frac{1}{2}]$$

where the fraction cleaved is calculated according to

$$\text{fraction cleaved}=\frac{\text{intensity digested bands}}{(\text{intensity digested band}+\text{ intensity undigested band})}$$

### Single cell cloning

Single-cell clones were obtained by limited dilution using conditions as reported elsewhere (Fernandes et al. 2012). An estimated five cells per well were introduced, and colony growth was supported by using conditioned media (supernatant of exponentially growing Sf9 cells) supplemented with 10% (v/v) fetal bovine serum (FBS) (Gibco). This setup was chosen to maximize the likelihood of obtaining single-cell colonies per well, accounting for expected cell loss due to stress during serial dilutions and plating, and to support the growth of single-cell clones following limited dilution. To confirm that colonies derived from a single cell, pictures of each well were taken after inoculation (day 0) and after colony growth (~ day 14–21), using a Cytation 3 Cell Imaging Reader (BioTek).

### Nanopore sequencing

Nanopore sequencing was used to confirm successful editing of the targeted DNA sequence. For genomic and cDNA sequencing, a 3000 bp and 1200 bp amplicon, respectively, were amplified from both wild-type and *Sf-Dronc* clones using primer pair 2 (Table S1) and then purified using AMPure XP-PCR beads (Beckman) according to the manufacturer’s instructions. Briefly, PCR products were mixed with magnetic beads in a 1:1 ratio and incubated for 5 min at room temperature. Next, the beads were pelleted using a magnetic rack (Biorad) and washed twice for 30 s with 70% ethanol. Residual ethanol was removed by air drying the beads, after which DNA was eluted from the beads using TE buffer. The purified PCR product was then collected and sent for Nanopore sequencing using Eurofin’s Genomic™ Linear Amplicon workflow. The acquired data was evaluated using the Epi2Me™ amplicon workflow and visualized using IGV™, using the *Sf-Dronc* amplicon amplified and purified from wild-type as the reference sequence.

### Gene expression analysis

Total RNA was extracted with High Pure RNA Isolation kit (Roche) according to the manufacturer’s instructions. RNA was quantified using a NanoDrop™ One/One^C^ (Thermo Fisher Scientific) and stored at −80 °C until further analysis. Reverse transcription was performed using Transcriptor High Fidelity cDNA Synthesis Kit (Roche) according to the manufacturer’s instructions with anchored-oligo (dT)18 Primer and 1 µg of RNA within a 40 µL reaction. Samples were diluted 1:5 within nuclease-free water (Gibco) and used alongside respective primers and LightCycler 480 SYBR Green I Master Kit (Roche). RT-qPCR reactions were performed in a LightCycler 480 Instrument II 384-well block (Roche) and quantification cycle values (Cts), and melting curves were determined using the LightCycler 480 Software version 1.5 (Roche). All data was analysed relative to the housekeeping gene Actin A1 (accession number HQ008727.1) and presented by employing the 2^−ΔΔCT^ method for relative expression analysis as fold change. Primers were designed to target the mutation site (with cDNA primer pair 1).

### Toxicity resistance assay

Responses to cellular stress were evaluated by MTT assay using Zeocin® (Invivogen). Briefly, 0.5 × 10^5^ cells were seeded per well and incubated until the cells were settled. Next, the supernatant was removed and replaced by twofold dilutions of Zeocin™ (0.001–4 mg.mL^−1^) in 100 µL Sf-900 II SFM media; non-treated cells were used as the control. Cells were incubated for 4 days at 27 °C, after which 0.05 mg thiazolyl blue tetrazolium bromide (MTT) (Abcam) was added per well. After allowing crystal formation for 4 h at 27 °C, the supernatant was removed, and the formed crystals were dissolved in dimethylsulphoxide (DMSO) (Sigma-Aldrich), by incubating for 15 min at room temperature. Finally, the absorbance at 570 and 690 nm (reference) was measured, and the IC50 value was determined by non-linear regression of normalized values using GraphPad Prism.

### Recombinant baculovirus amplification and storage

Recombinant baculoviruses (rBAC) containing transgenes of interest were amplified as described elsewhere (Vieira et al. 2005). Briefly, insect Sf9 cells were infected at a concentration of 1 × 10^6^ cell.mL^−1^ using an MOI of 0.1 plaque-forming units per viable cell (pfu.cell^−1^). When cell viability reached approximately 80%, rBAC was harvested by performing two centrifugation steps (200 × g for 10 min at 4 °C and 2000 × g for 20 min at 4 °C). The clarified supernatant was then aliquoted and stored at 4 °C until further use.

### Production of rAAV, iVLPs, and PfRipr5

The established *Sf-Dronc* knockout clones were assessed for the production of three different biologics: a recombinant adeno-associated virus (rAAV), a virus-like particle displaying the influenza M1 protein (hereby named iVLPs), and a recombinant antigen against malaria (named PfRipr5).

rAAV carrying a GFP transgene expression cassette was produced using two rBACs, namely rBAC-REP/CAP (carrying AAV2 rep and cap genes as established in-house using Addgene plasmid #65214) (Smith et al. 2009; Pais et al. 2019) and rBAC-GFP (incorporating a gfp transgene flanked by AAV serotype 2 inverted terminal repeats as reported previously) (Virgolini et al. 2023). iVLPs were produced using one rBAC, carrying both the influenza M1 (from A/California/06/2009 H1N1 strain) and the HA (from A/Brisbane/59/2007 strain) genes; this rBAC was generated as described elsewhere using FlashBac™ (Correia et al. 2025). PfRipr5 was expressed using one rBAC as described elsewhere (Correia et al. 2022).

Production of all three biologics was initiated by infecting Sf9 and *Sf-Dronc* knockout clones at a cell concentration of infection (CCI) of 2 × 10^6^ cell.mL^−1^, with a multiplicity of infection (MOI) of 0.1. As two rBACs were required for rAAV production, both viruses were added with an MOI of 0.05 pfu.cell^−1^. Samples for cell concentration, viability, and product titre assessment were taken approximately every 24 h.

### Analytics

#### Cell concentration and viability

The trypan blue dye exclusion method was used to determine viable cell concentration and cell viability (Tennant, 1964), utilizing automatic counting by the Cedex HiRes Analyzer (Roche) or the ViCell Blue (Beckman Coulter).

#### rAAV quantification

Samples for rAAV titre determination were processed as described elsewhere (Pais et al. 2019) and stored at −80 °C until further analysis. Intracellular samples were digested as previously reported (Virgolini et al. 2023). Intracellular rAAV viral genomes (VG) were estimated by real-time quantitative PCR (RT-qPCR), as described previously with primers and probes in Table S1 (Pais et al. 2019). Total particles using ELISA were performed using the AAV2 Xpress kit by Progen™ according to the manufacturer’s instructions.

#### Hemagglutination assay

HA titre was determined by hemagglutination assay, as described elsewhere ^4^. Briefly, samples were serially diluted 1:1 in 1 × PBS (Corning), using V-bottom plates (Sarstedt). Next, samples were mixed 1:1 with 1% chicken erythrocytes (Rockland), after which they were incubated for 30 min at 4 °C. After visual inspection, the HA titre was estimated as being the inverse of the highest sample dilution with no detectable hemagglutination.

#### Western blot

Expression of PfRipr5 was assessed in culture supernatants by western blot, as shown elsewhere (Correia et al. 2022). iBright™ (Thermofisher) imaging system was used to take pictures of the resulting membranes, while GelAnalyzer™ was utilized for densitometry analysis of protein bands.

#### Baculovirus titration

Recombinant baculovirus was titrated using the MTT assay as described elsewhere (Mena et al. 2003; Roldão et al. 2009).

## Results

### Ribonucleoprotein delivery in insect Sf9 cells

Given the current state of the art and the lack of publications describing generating gene knockout (KO) Sf9 cell line clones in the industry, we recognized the need for a more efficient and thus faster CRISPR-Cas9 based pipeline. For this, CRISPR-Cas9 ribonucleoprotein complexes (RNP) delivery was evaluated using various transfection methods and compared to the reported plasmid-based delivery strategy described previously to be successful in the insect Sf9 cell line (Mabashi–Asazuma).

**Fig. 1:**
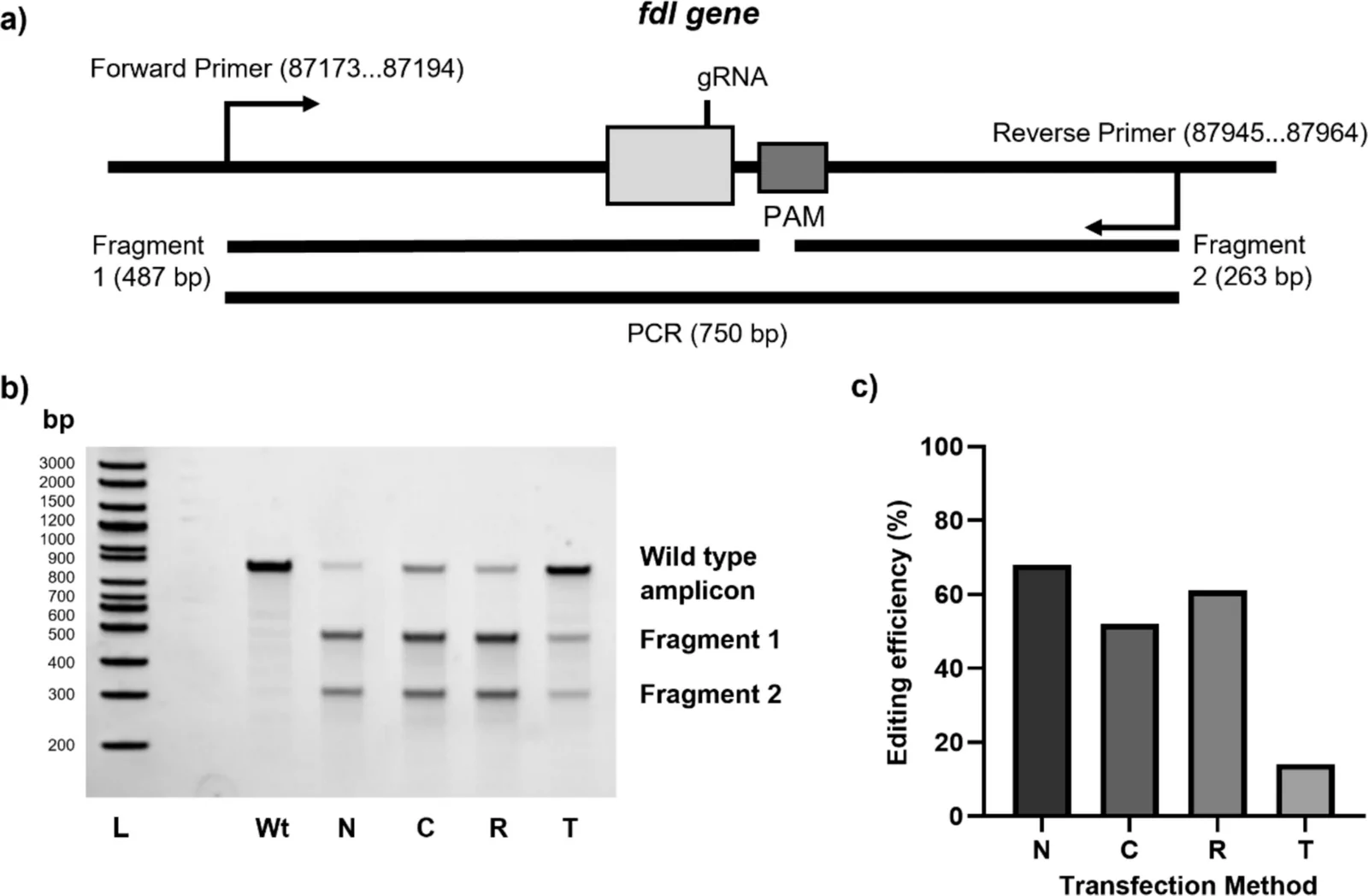
Evaluation of editing efficiencies using different CRISPR-Cas9 ribonucleoprotein (RNP) complex delivery methods: **a** Target region representation of genome and expected fragments according to gRNA target region; **b** Evaluation by T7 assay of edited amplicons derived from PCR, with different delivery strategies represented; N, Nucleofection™; C, Cellfectin™; R, RNAiMAX™; T, TransIT™; Wt, wild type; L, molecular weight ladder; **c** Efficiency evaluation across delivery methods

To assess the editing efficiency of different CRISPR-Cas9 RNP delivery methods, the previously reported *fdl* gene was targeted, using an established sgRNA sequence from a previous report (Fig. 1a and Table S1) (Mabashi-Asazuma and Jarvis 2017). RNP complexes were generated and delivered to Sf9 cells using lipofection or electroporation as DNA delivery methods, deemed suitable due to the negative charge of RNP complexes. The percentage of edited *fdl* to wild-type amplicon was assessed by T7 assay. All selected commercial lipofection reagents (TransIT™, T; RNAiMAX™, M; and Cellfectin™, C) allowed successful delivery of RNP complexes, with the highest editing efficiency observed for RNAiMAX™ (Figs. 1b–c). Electroporation by Nucleofection™ resulted in a similar editing efficiency value to the best lipofection reagent RNAiMAX™ (68% vs. 61%, respectively), showcasing the potential of this method for RNP complex delivery. Overall, these results are significantly higher than those observed previously (as calculated by densitometric analysis of published gel) (Mabashi-Asazuma and Jarvis 2017). Due to its lower cost and better scalability, lipofection using RNAiMAX™ was selected as the delivery method of RNP complexes for further experiments.

### Targeting Sf-Dronc

The applicability of the method was demonstrated by successfully deleting the *Sf-Dronc* gene. Apoptosis has been shown to play a significant role in the lytic IC-BEVS system, decreasing cell lifespan and thereby affecting product titres and/or quality (Hong et al. 2022). However, since caspases are present within cells in an inactive state, silencing caspases (using methods such as dsRNA) has a limited effect, allowing caspases to contribute to apoptosis induction (Huang et al. 2013). Initiator caspases, such as *Sf-Dronc*, a homologue to the *Drosophila dronc* gene and recently identified to be differentially expressed during infection processes, have not been studied extensively (Virgolini et al. 2023). With the limitation of the lytic nature of the BEVS system, knocking out caspases has the potential to enhance product titre and/or quality of ATMPs by avoiding the accumulation of uncleaved (or inactive) caspase within the cytoplasm and therefore caspase activation.

**Fig. 2:**
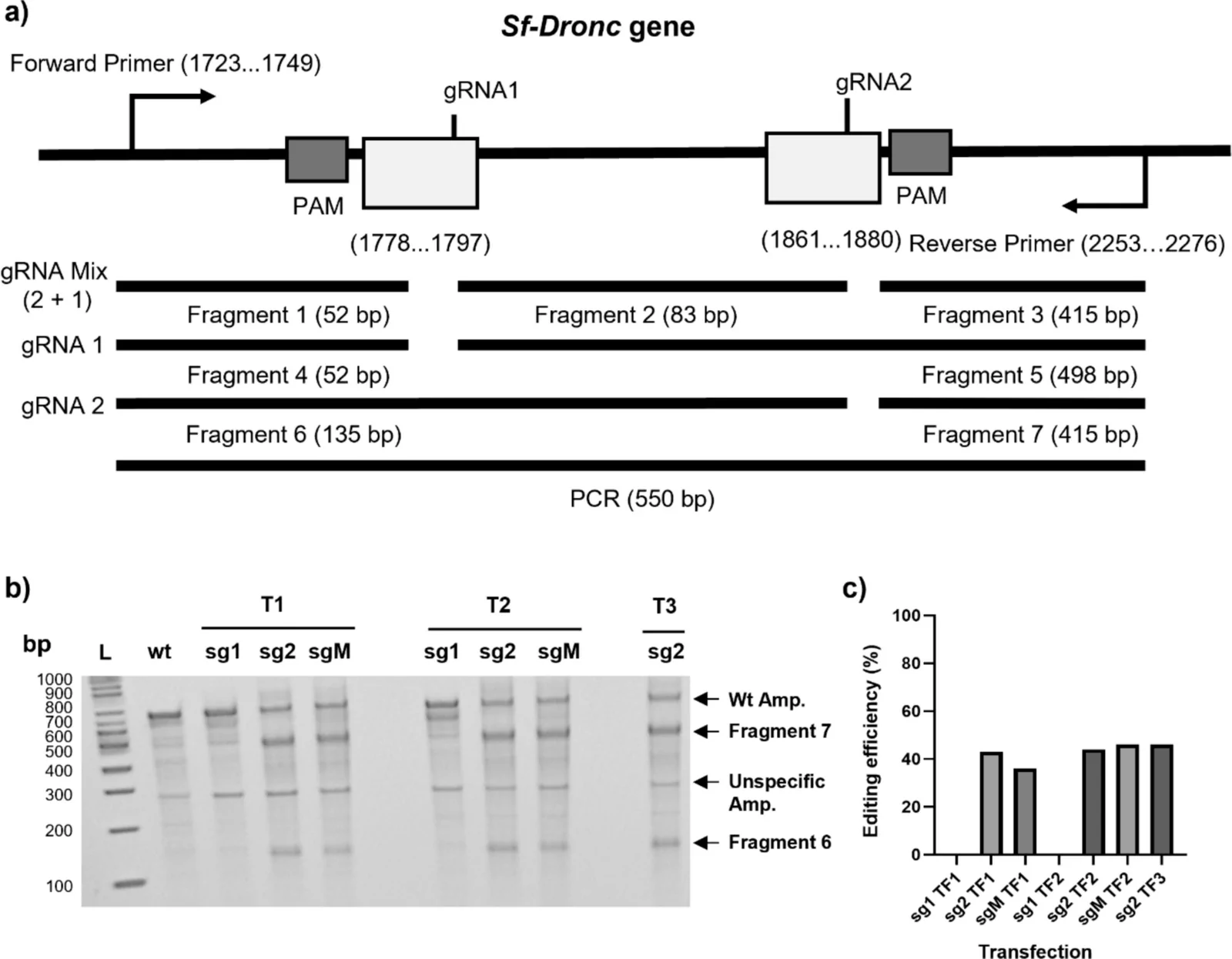
Evaluation of *Sf-Dronc* knockout: **a** Target region representation of gene and expected fragments according to gRNA target region; b Evaluation by T7 assay of edited amplicons derived from PCR, where T1, T2, and T3 correspond to first, second, and third transfection and sg1, sg2, and sgM correspond to delivery of gRNAs in a single or mixed format; **c** Efficiency evaluation across transfections

Two gRNAs targeting the second exon of the *Sf-Dronc* gene were designed and transfected individually (sg1 and sg2) or as a mixture (sgM), using the previously established protocol (Fig. 2a). Initial assessment of these gRNAs indicated decreased editing performance compared to *fdl*, with values below 45% for both sg2 and sgM and with sg1 showing no target editing efficiency (Figs. 2b–c). A second and third round of transfection were performed to further enhance gene editing efficiency and thus maximize the chances of isolating a full knockout mutant. No significant improvements in the percentage of edited target DNA were observed (Figs. 2b–c), showing subsequent transfections for the knockout of this gene are not required.

### Sf-Dronc knockout clones screening

**Fig. 3:**
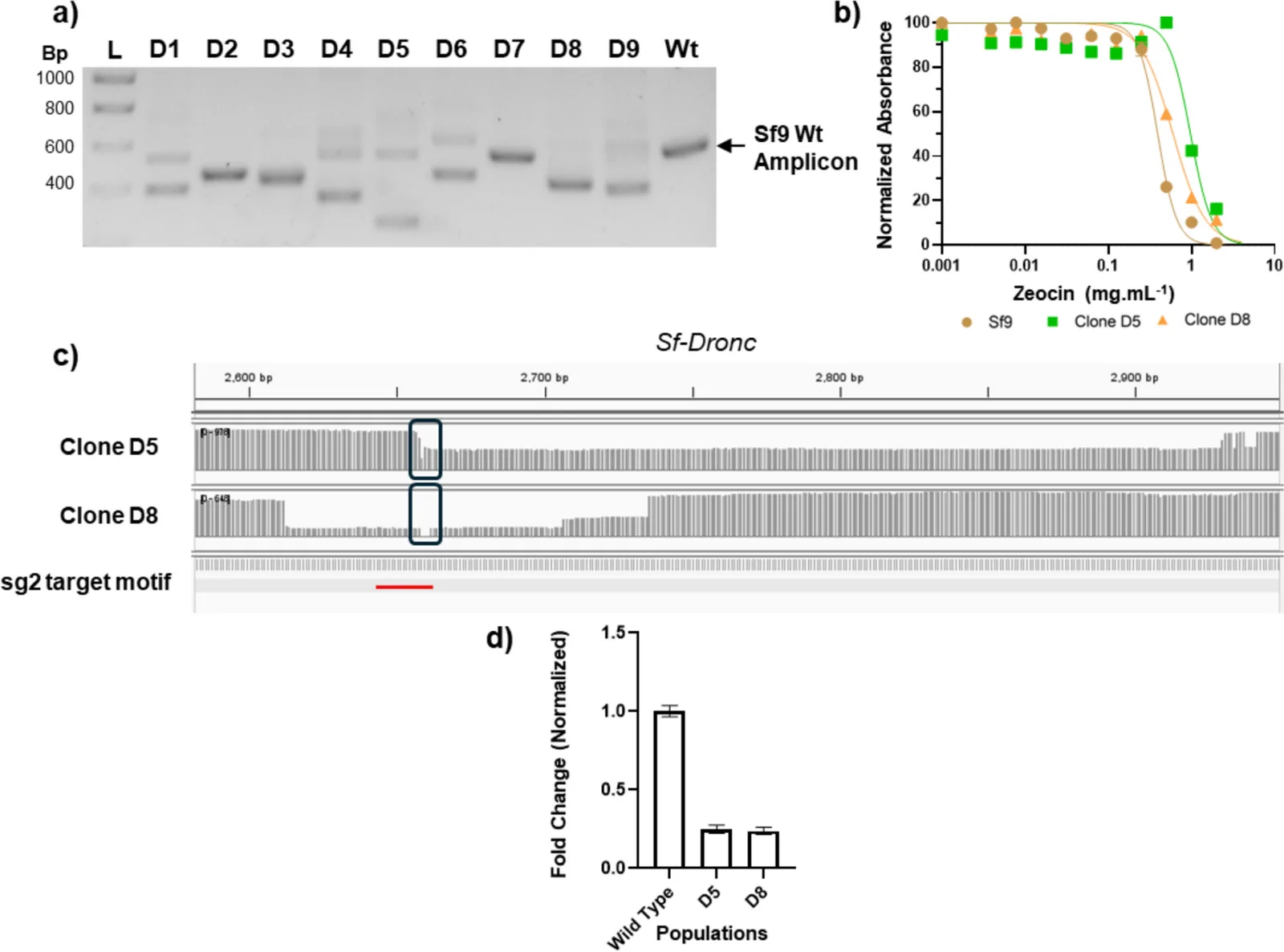
Knockout of *Sf-Dronc* gene: **a** evaluation by colony-PCR of amplicon size observed in clones D1–D9 and comparison with wild type (Wt) amplicon; **b** Characterization of cellular response to apoptosis induction by Zeocin™ as assessed by MTT assay and evaluated using GraphPad Prism™; results are shown as average of eight biological replicates (*n* = 8), error bars represent standard deviation; **c** Amplicon coverage of mutants D5 and D8 (genomic sequence) when comparing to amplicon present in wild type cells as seen using IGV™ to visualize Nanopore sequencing data; box highlights the region of interest, featuring the target motif in red; **d** Relative expression profile measured by RT-qPCR of *Sf-Dronc* transcripts in mutant populations when compared with wild-type, normalized to Actin-b

Limiting dilution was used to establish single-cell knockout clones derived from cells that were transfected with RNP complexes and sgRNA targeting the Dronc gene. Single cells were allowed to reach a suitable cell number for colony PCR screening and subsequent scale-up. From the nine selected clones, eight showed a visible shift in the amplicon size of the *Sf-Dronc* sequence, thus suggesting a deletion of the target sequence (Fig. 3a). Notably, most of the clones exhibited amplicon bands below the wild-type (below 400 bp), indicating that different alleles suffered different mutations (e.g. frameshift, large deletions). Two clones (D5 and D8) were selected for phenotypic screening after assessing clonality to evaluate their cell growth kinetics both in the absence and presence of stress induced by the apoptotic agent Zeocin™. This intercalating agent is responsible for inducing double-stranded DNA breaks, being directly connected to an apoptotic response. In the absence of Zeocin™, the *Sf-Dronc* knockout clones showed a reduced cell growth rate and peak cell concentration compared to wild-type Sf9, exhibiting an earlier onset of cell viability drop (Figure S2). More importantly, in the presence of Zeocin™, both clones showed a delayed and/or decreased onset of apoptotic response, as validated in the MTT assay, with the IC50 values estimated for clones D5 and D8 (0.97 ± 0.07 and 0.60 ± 0.03 mg.mL^−1^, respectively) being higher than the wild-type Sf9 (0.39 ± 0.02 mg.mL^−1^) (Fig. 3b). To confirm that both clones have completely knocked out the *Sf-Dronc* gene, target amplicons were purified and assessed using Nanopore sequencing (Fig. 3c). Compared to the wild-type, both clones showed deletion of a large fragment of the *Sf-Dronc* gene within the genome in some of the alleles. To confirm editing at the transcript level, RNA was extracted, and cDNA was quantified through qPCR as well as sequenced using Nanopore. While sequencing proved that the deletions observed in the genomic sequence correspond to those observed in the mRNA (Figure S6), with small mutations detected within the target area, RT-qPCR of the target region within the mRNA shows that expression levels of the *Sf-Dronc* transcripts are downregulated when compared with the wild-type population (Fig. 3d), with both clones showing approximately a 75% reduction in transcript levels. These results suggest that while not full knockouts, both mutants represent hypomorph populations with downregulation of functional *Sf-Dronc*.

### Assessing Sf-Dronc knockout clones for production of rAAV, iVLPs, and PfRipr5

**Fig. 4:**
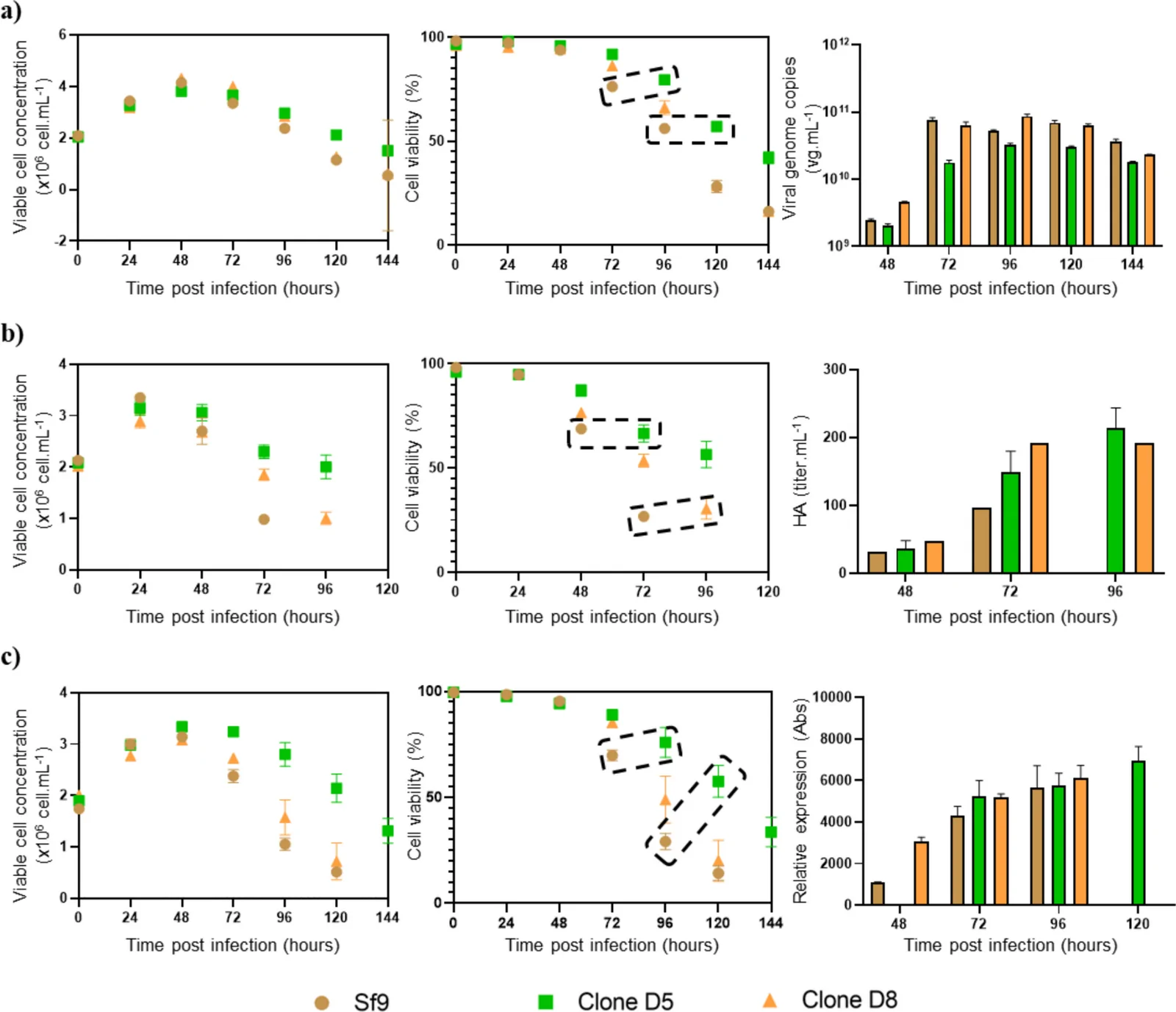
Kinetics and productivity during infection of knockouts and wild type using recombinant baculoviruses: **a** production of recombinant adeno-associated viruses; **b** production of virus-like particles displaying influenza-HA; **c** production of a PfRipr5, a malaria subunit, with viable cell concentration on the right, cell viability in the centre, and productivity on the right. Viability drop delay highlighted by dashed boxes. For **a–c**: results represent the average of three biological replicates (*n* = 3); error bars represent standard deviation

To evaluate whether the established clones exhibit an improved production phenotype (i.e. if a delayed onset of cell viability drop contributes to increased product titres), three different biologics were expressed: rAAV, iVLPs, and PfRipr5 (Fig. 4). Overall, Sf-Dronc knockout clones showed a delayed onset of cell death of approximately 24 h when compared to wild-type Sf9 cells; this difference was particularly pronounced in cells expressing iVLP and PfRipr5. Among the two clones evaluated, clone D5 showed the slowest decrease in cell viability upon infection. Regarding product titres, while no significant increase in intracellular rAAV titres and PfRipr5 expression was observed (Figs. 4a–b), an > twofold increase in iVLP titres was achieved (Fig. 4c). These results suggest that the established Sf-Dronc knockout clones have a product-related improvement in productivity compared to wild-type Sf9 cells.

## Discussion

CRISPR-Cas9 is a powerful tool to establish targeted and accurate gene knockouts (Doudna and Charpentier 2014) and has shown great potential in increasing product titres and/or quality in producer cell lines (Raab et al. 2019; Ley et al. 2019). Previous work of Mabashi-Asazuma and Jarvis (2017) reports the use of a plasmid-based method for the delivery of the necessary genes for CRISPR-Cas9 in insect cells. However, results achieved showed limited efficiency, mostly as a consequence of the lack of knowledge surrounding efficient promoters in insect cells (Mabashi-Asazuma and Jarvis 2017). The discovery of more efficient promoters has been the object of recent studies, leading to some advancements which can better leverage the plasmid-based delivery into the editing of insect cells (Miyata et al. 2019). In our study, we developed a CRISPR-Cas9 delivery method using ribonucleoprotein (RNP) complexes which have shown to be efficient, resulting in higher editing efficiencies compared to previously established plasmid-based delivery methods (Mabashi-Asazuma and Jarvis 2017). As such, this pipeline has the potential to accelerate genetic engineering applications in the insect cell-baculovirus expression vector system (IC-BEVS), enabling the rapid and effective generation of mutant phenotypes.

Engineering insect Sf9 cells presents two major challenges: a biological constraint arising from their mixed diploid/tetraploid nature (Jarman-Smith et al. 2004) and a technical limitation associated with their low survival as single‑cells that complicates conventional cloning approaches (Vidigal et al. 2013; Ma et al. 2019). Regarding the biological constraint, the simultaneous deletion of all alleles and the subsequent identification of complete knockout clones is considered challenging, prompting the need for tools capable of generating high frequencies of editing in a population and reliably determining the resulting mutations. While standard Sanger sequencing is sufficient for detecting mutations in bi-allelic populations, it is not feasible for resolving the complex mixture of alleles typically present in Sf9 cells. Techniques such as Nanopore sequencing or RT‑qPCR are more suitable for characterizing heterogeneous editing outcomes and assessing the extent of gene disruption. Nanopore sequencing shows that mutations and large deletions are present in the genome of the sequenced clones and that the same mutations found in the target region can be seen in the cDNA. These mutations impacted gene expression, evidenced by the reduced levels of Sf-Dronc transcripts in mutant populations compared to wild-type, as measured by RT-qPCR. Importantly, the transcripts that remain detectable likely correspond to hypomorphic alleles carrying some of them indel mutations, resulting in non-functional Sf-Dronc expression. Although we were unable to directly assess protein abundance due to the lack of a suitable antibody, our data suggest that the expression of the functional initiator caspase SfDronc is markedly diminished due to the indels generated. Regarding the technical constraint, Sf9 cells display very low survival under strict ≤ 1 cell‑per‑well limiting‑dilution conditions. To accommodate this limitation, the cloning step in this workflow was adapted to the biological characteristics of Sf9 cells by using a slightly higher seeding density together with imaging‑based monitoring to ensure that the selected colonies originated from single cells. Although suitable for R&D‑focused gene‑editing workflows, stricter ≤ 1 cell‑per‑well cloning procedures remain the standard for clinical or regulatory‑grade cell line development. Future implementations of this workflow, particularly for applications requiring formal clonality assurance, will incorporate such stringent single‑cell cloning procedures despite the associated viability challenges in Sf9 cells.

In the IC-BEVS, production is severely limited by the lytic nature of the system. Despite the viral expression of anti-apoptotic genes, we previously confirmed the activation of the apoptotic pathway during baculovirus infection (Virgolini et al. 2023). As such, targeting the apoptosis pathway might result in insect cell lines showing delayed onset of cell viability drop upon baculovirus infection, potentially improving product titres and/or quality. To date, effector caspases like caspase-1 have been investigated through inhibition, knockout, or both. These studies have resulted in mutant clones exhibiting enhanced apoptosis resistance under non-infectious conditions, though they have yielded inconsistent outcomes in improving viability during infectious processes (Hebert et al. 2009; Lai et al. 2012; Huang et al. 2013; Yang et al. 2016; Zhang et al. 2018, 2021b; de Malmanche et al. 2022). Initiator caspases, such as Sf-Dronc, which are found downregulated during baculovirus infection in one of our previous studies (Virgolini et al. 2023), have not been extensively explored. These molecules are known to activate effector caspases (i.e. caspase-1) in insect cells (Meier et al. 2000; Huang et al. 2013) and thus might prove more efficient in preventing apoptosis initiation during infection processes. Indeed, the dronc knockout clones established herein highlighted a delayed onset of cell viability drop during baculovirus infection, likely correlated with improved apoptosis resistance and similar to what has been achieved previously using RNAi gene silencing (Huang et al. 2013).

Inhibiting apoptosis has been associated with improving product titres and/or quality in IC-BEVS (Steele et al. 2017; Zhang et al. 2018, 2021b) and other expression systems (e.g. Chinese Hamster Ovary cells) (Grilo and Mantalaris 2019). However, a recent study in IC-BEVS could neither confirm a delayed onset of cell viability drop during infection nor improved product titres when knocking out or inhibiting caspase-1, hypothesizing that previous improvements were product-dependent and/or associated with off-target effects (de Malmanche et al. 2022). In this study, the Sf-Dronc knockout clones established successfully improved cell viabilities after infection; while this enabled a > twofold increase in iVLP production compared to wild-type populations, no improvements were observed for rAAV2 or PfRipr5 titres.

This variability in phenotype may reflect the limitations of targeting apoptosis alone to enhance production processes; nonetheless, it may also underscore the influence of two key factors: population heterogeneity prior to single-cell cloning and product–process-specific constraints. Regarding population heterogeneity, single-cell cloning has long been critical in biotechnological applications, with regulatory agencies requiring proof of clonality for cell lines used in biopharmaceutical production. Monoclonality ensures consistent population behaviour, product quality, and genetic stability (Yang et al. 2022; Boulenouar et al. 2023). In the context of CRISPR-Cas9 gene editing, monoclonality prior to transfection minimizes genetic variability among generated mutants, while post-transfection it enables the selection of clonal populations carrying complete knockout mutations with uniform deletions (Giuliano et al. 2019). Although our current workflow includes single-cell cloning after transfection, initiating editing from a genomically homogeneous population could further reduce variability and simplify downstream characterization. For future applications of the CRISPR-Cas9 system reported herein, assuring monoclonality prior to transfection could facilitate screening and interpretation of phenotypic outcomes and enhance the industrial applicability of engineered cell lines. To achieve this, cell line engineering should focus on using wild-type clone-derived populations that demonstrate genomic stability across passages and on identifying markers associated with this trait (Wurm and Wurm 2017; Cordova et al. 2024).

Regarding product–process-related limitations, rAAV2 is an intracellular product, and, as such, cell productivities might be restricted by the cell machinery and the cell’s capacity to produce and accumulate intracellular proteins, and/or molecular precursors. Additionally, the low multiplicity of infection (MOI < 1 pfu/cell) used for rAAV2 production requires baculovirus replication and release from insect cells, events that may be impacted/hindered by the mutation introduced in the cell. Further evaluations and optimization of infection conditions (e.g. different MOIs) are needed to assess the potential of *Sf-Dronc* knockout clones in improving rAAV2 titres. For iVLP production, the only condition used was one that was previously optimized for Sf9 cells, and indeed in this case, it was possible to observe not only a larger variance in the viability profiles of the knockout populations, but also a positive impact in productivity, suggesting a product-specific benefit.

## Conclusion

This study presents a novel and efficient CRISPR-Cas9–based gene editing pipeline for Sf9 insect cells, serving as a proof of principle for enhancing genetic engineering in insect cell lines. The method successfully delivered a ribonucleoprotein (RNP) complex consisting of Cas9 and guide RNA into Sf9 cells, significantly improving previous systems. Using the system, a new cell line was established showing improved apoptosis resistance, as highlighted by a delayed onset of cell viability drop during baculovirus infection. While this phenotype could not lead to enhanced rAAV2 nor PfRipr5 product titres, iVLP production was positively impacted. Overall, this study further enhances genetic engineering applications in the industrial relevant Sf9 insect cell line, providing a fast and efficient CRISPR-Cas9 gene knockout pipeline that can be leveraged to improve insect cell phenotypes towards enhanced protein expression.

## Supplementary information

Below is the link to the electronic supplementary material.

ESM 1 (PDF 1.80 MB)

## Data Availability

The sensitive nature of some of the reagents used in this study (e.g. cell lines, plasmids, baculoviruses and antibodies) denotes that they are only readily available to the author’s institutions staff for R&D purposes. For external researchers, approval of reagents may be obtained via email addressed to the corresponding author.
