# Supplementary material for: An improved CRISPR-Cas9 protein-based method for knocking out insect Sf9 cell genes
Source: Appl Microbiol Biotechnol. 2026 Jan 26;110:42. doi: 10.1007/s00253-026-13722-3 (PMC12847097; doi:10.1007/s00253-026-13722-3)
Supplement: Supplementary file 1 [file 253_2026_13722_MOESM1_ESM.pdf]

An improved CRISPR-Cas9 protein-based method for knocking out insect Sf9 cell genes

Miguel Graça[1,2], Nikolaus Virgolini[1,2], Ricardo Correia[1,2], Jose Escandell[1,2], António Roldão[1,2]

[1] iBET, Instituto de Biologia Experimental e Tecnológica, Apartado 12, 2780-901 Oeiras, Portugal
[2] Instituto de Tecnologia Química e Biológica António Xavier, Universidade Nova de Lisboa, Av. da República, 2780-157 Oeiras, Portugal

Corresponding author: aroldao@ibet.pt (+351214469418)

Figure S1 Images acquired using Cytation 3™ at day zero of inoculation and after identification of colonies (clones D5 and D8)

D5

D8

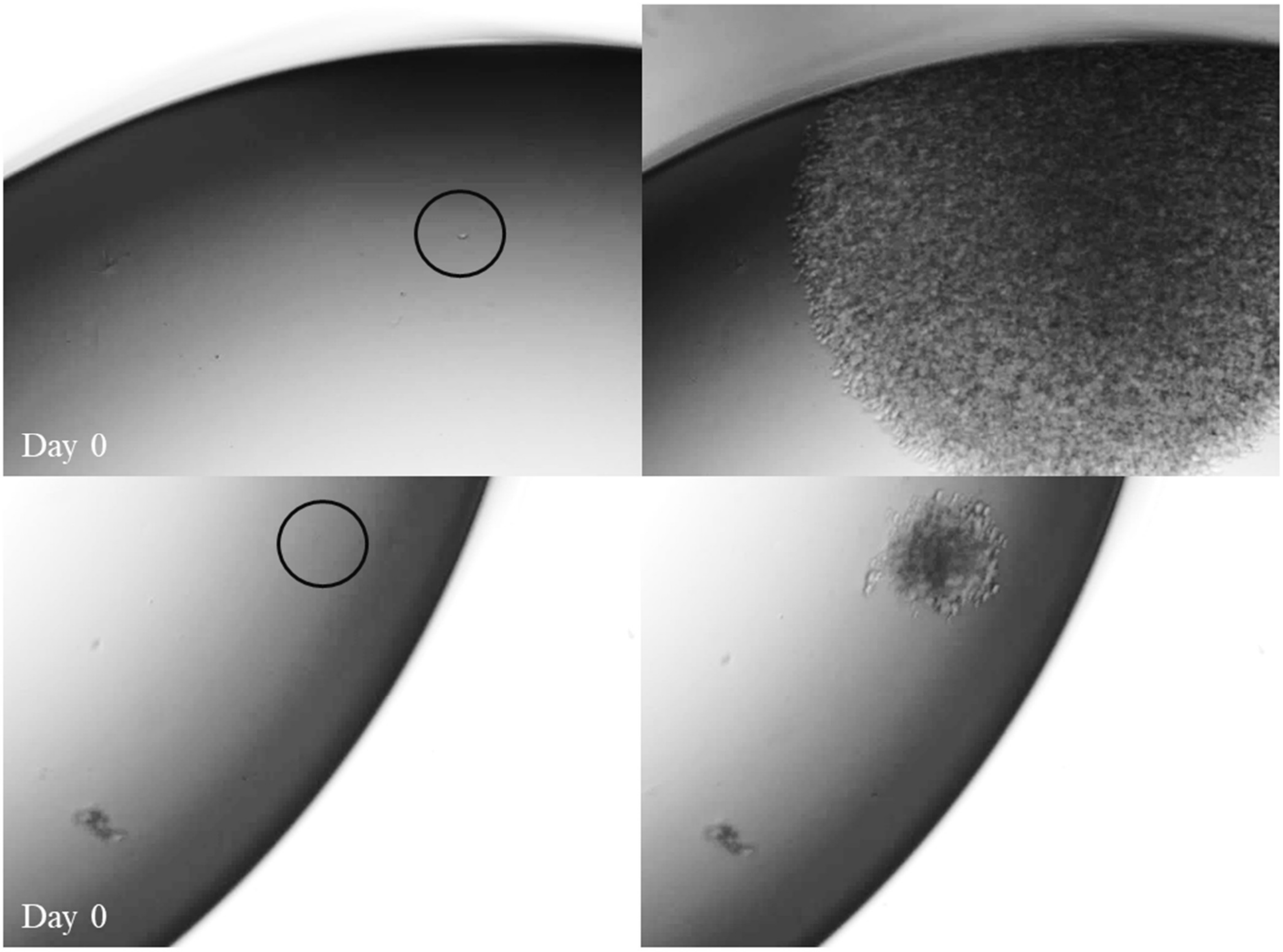

Figure S2 Assessment of kinetic profiles of knockout and wild type populations; results represent the average of three biological replicates (n = 3), error bars represent standard deviation

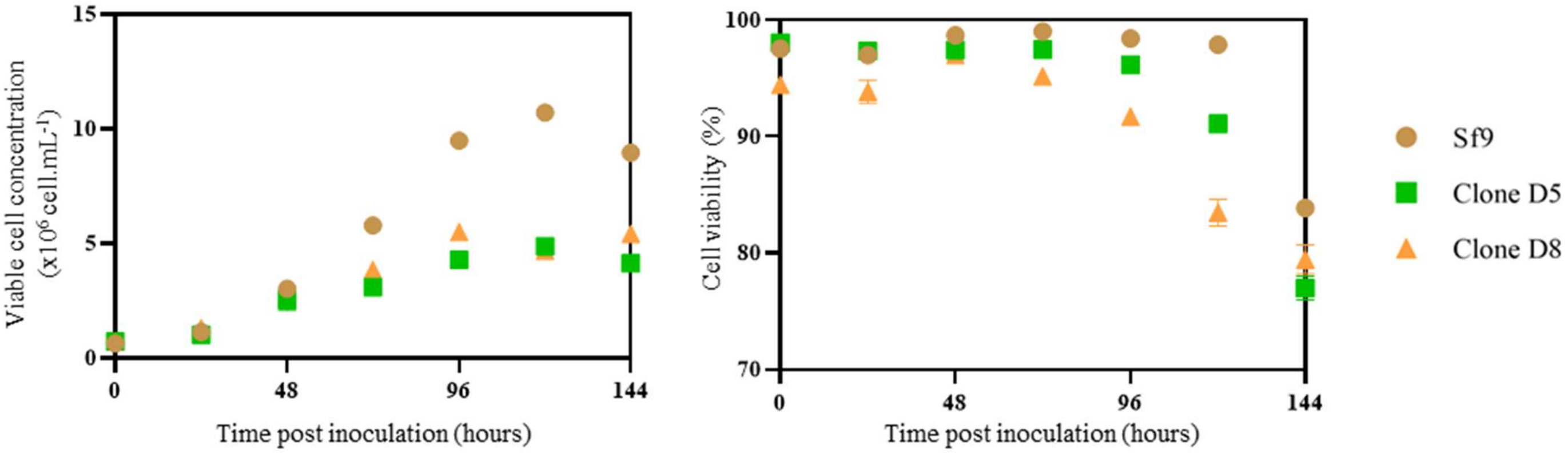

Figure S3 Evaluation by T7 assay of edited amplicons derived from PCR, with (-) representing wild type control, and transfection methods: N – Nucleofection™, C – Cellfectin™, R – RNAiMAX™ and T – TransIT™ (digestion of Nucleofection™ product and wild type control repeated)

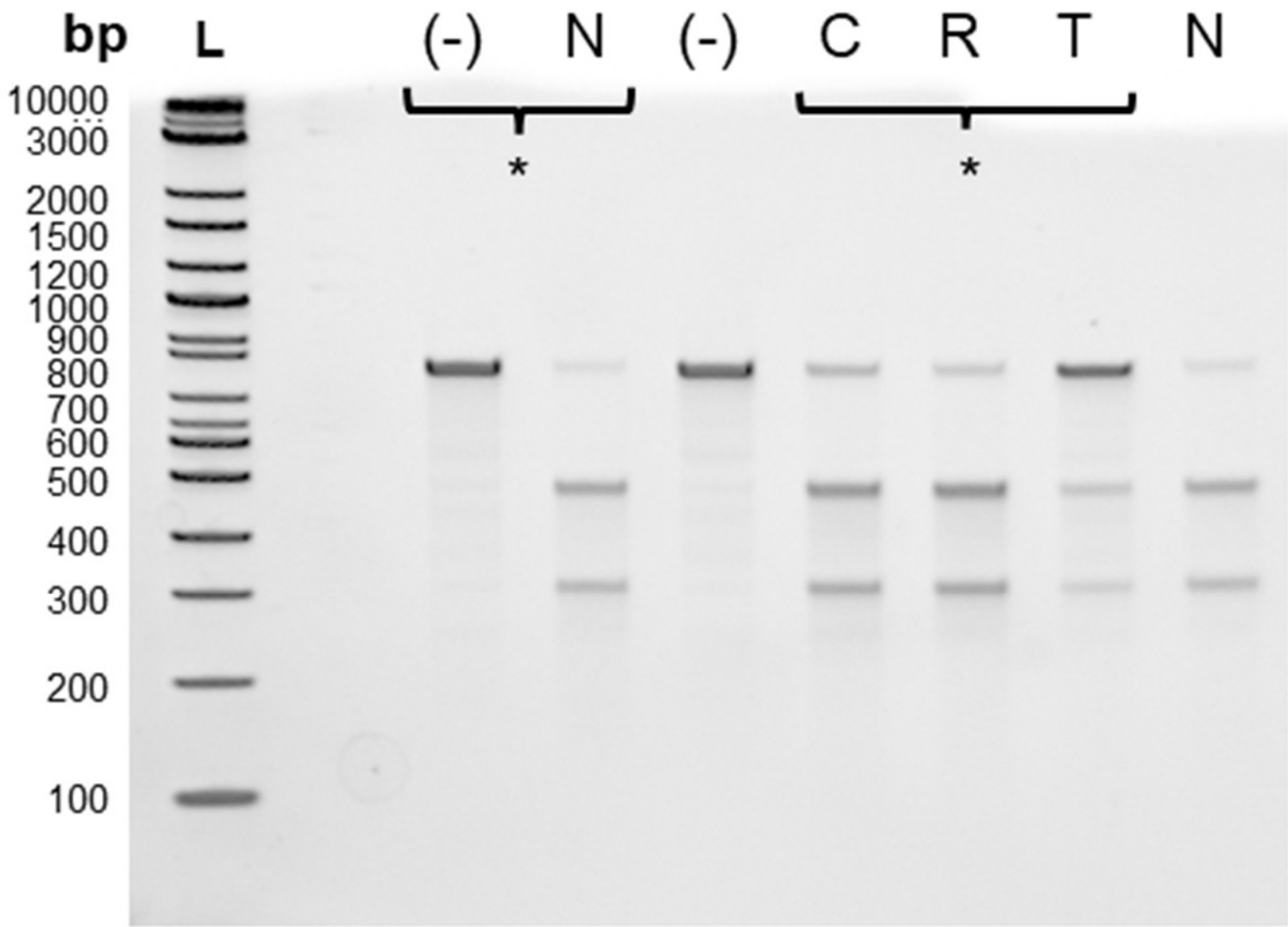

* Shown in manuscript

Figure S4 Evaluation by T7 assay of edited amplicons derived from PCR, where T1, T2 and T3 correspond to first, second and third transfection and sg1, sg2 and sgM correspond to delivery of gRNAs in a single or mixed format

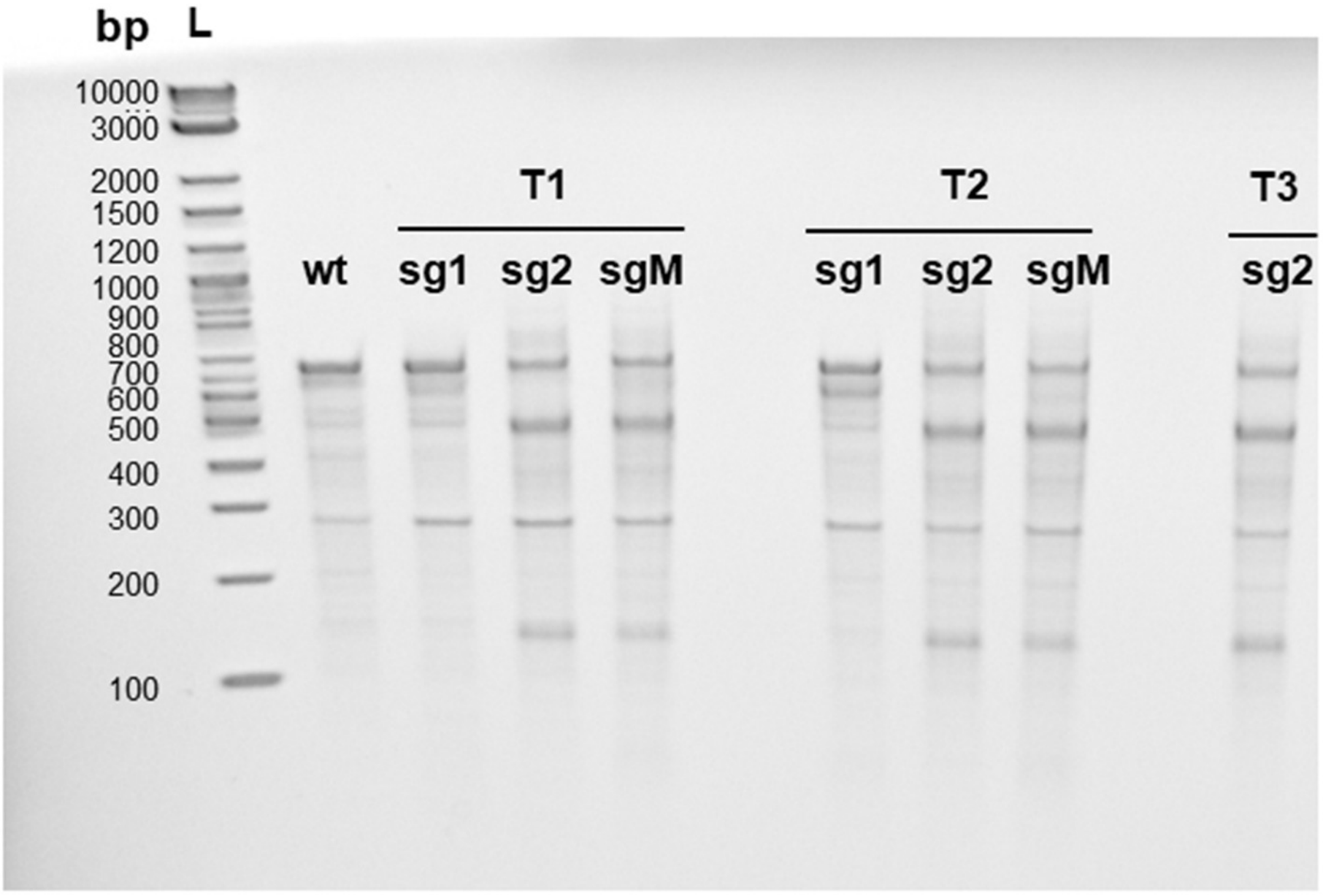

Figure S5 Evaluation by colony-PCR of amplicon size observed in clones D1-D9 and comparison with wild type (Wt) amplicon

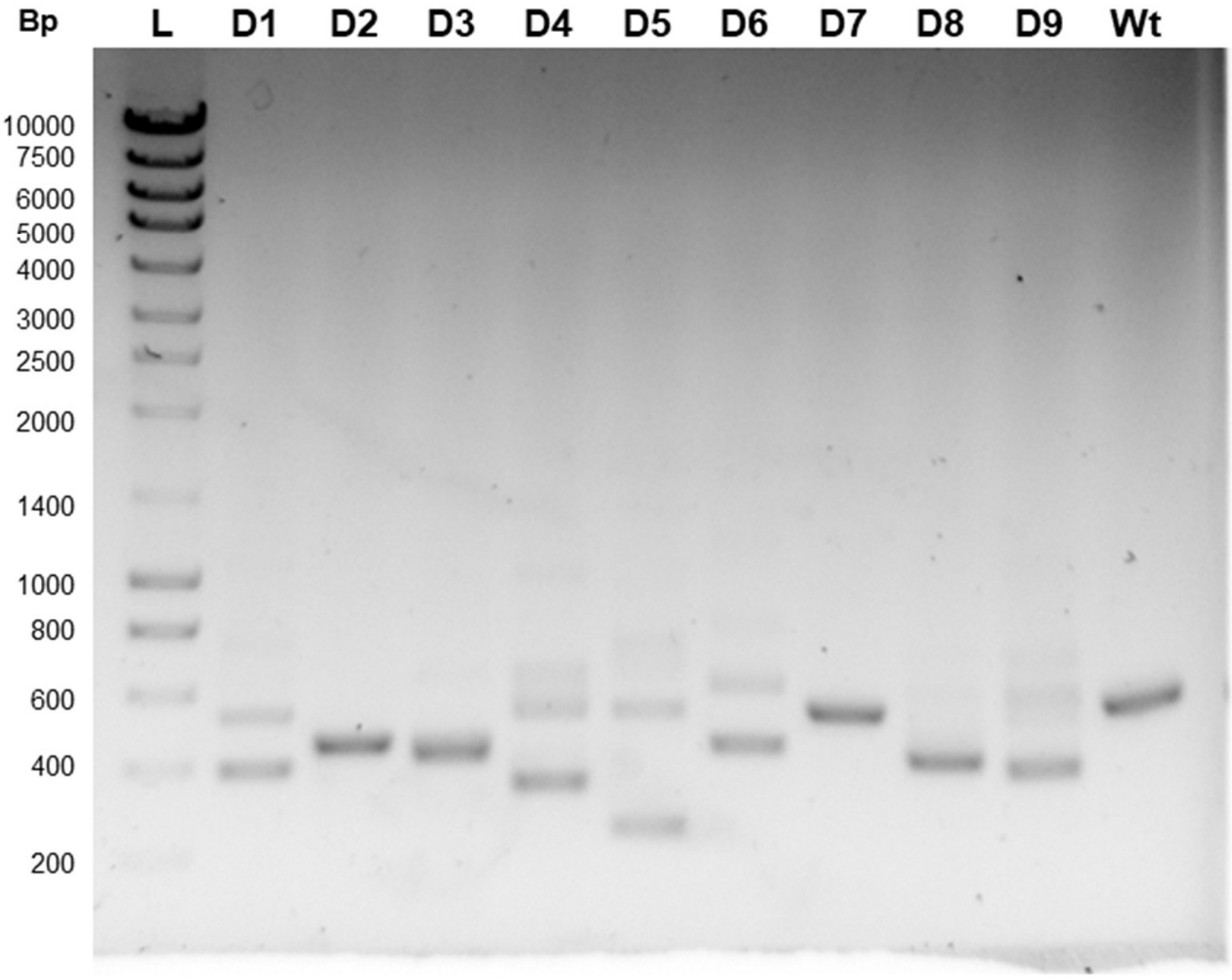

Figure S6 Amplicon coverage of mutants D5 and D8 (cDNA sequence) when comparing to amplicon present in wild type cells as seen using IGV™ to visualize Nanopore sequencing data; box highlights the region of interest, featuring the target motif in blue.

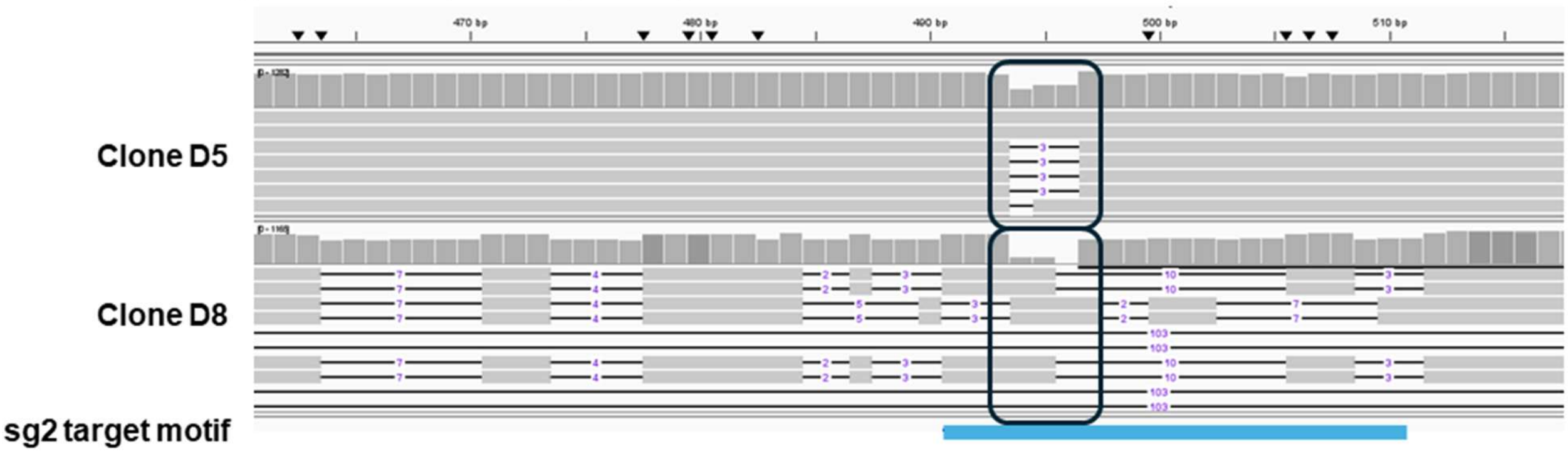

Figure S7 Relative expression of PfRipr5 between each cell population (wild-type and clone D1) at different times post infection (hpi: hours post infection), with each time point represented by three different biological replicates

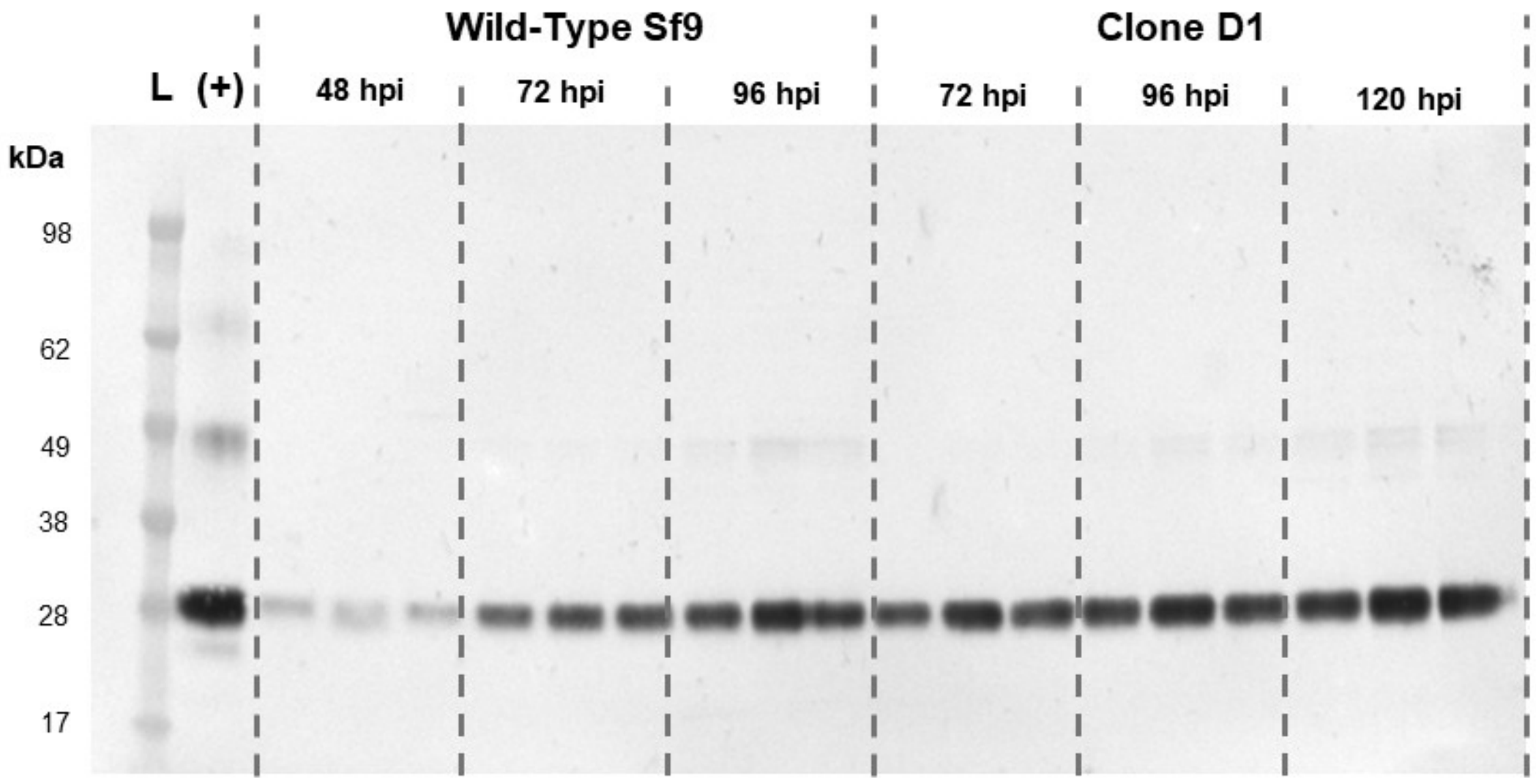

Figure S8 Relative expression of PfRipr5 using clone D1 throughput different times post infection (hpi: hours post infection), with each time point represented by three different biological replicates

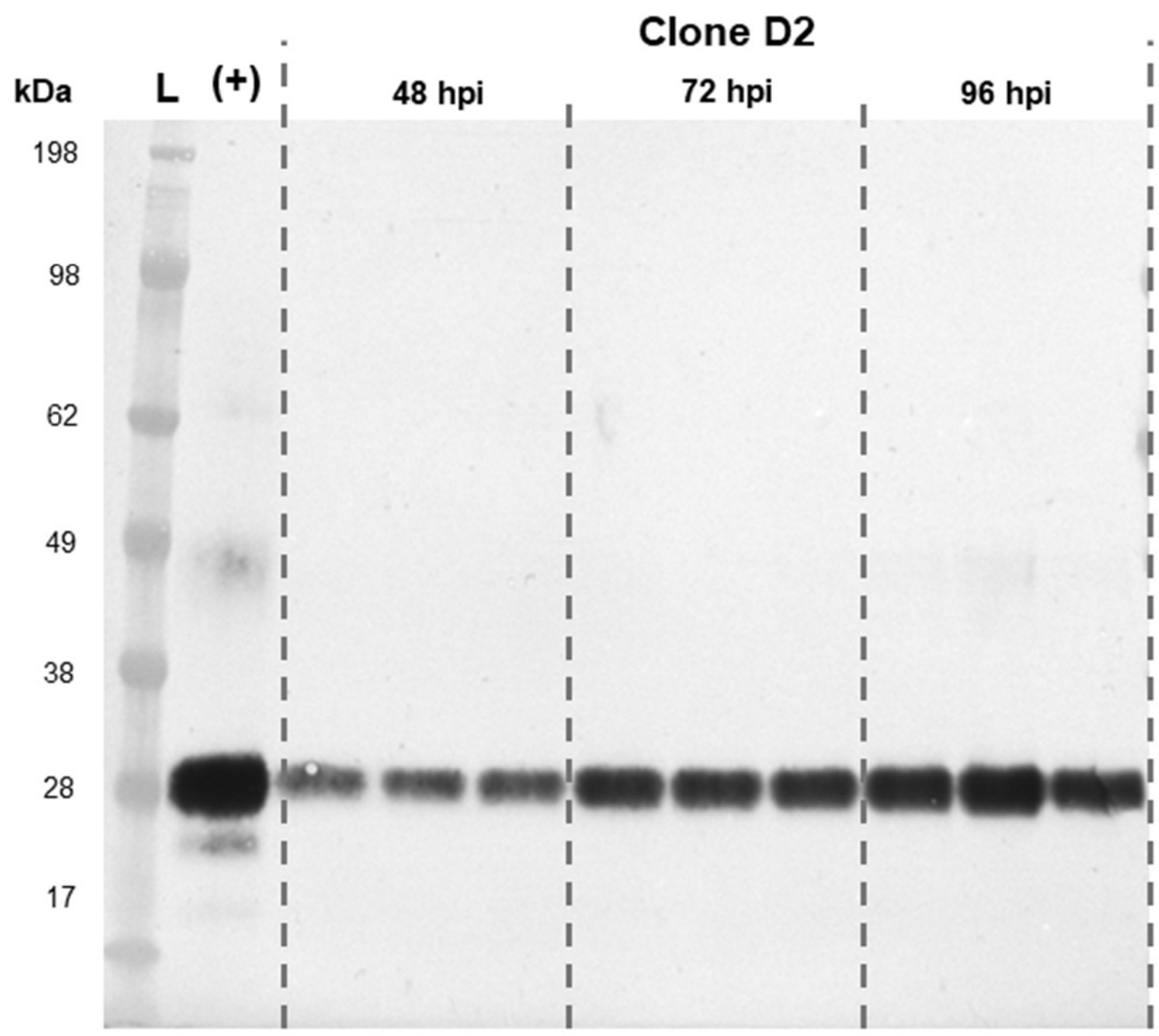

Table S1 The sgRNAs, amplification and RT-qPCR primers used in this study

Name	Sequence (5' → 3')	Type
**Fdl sg** *	ACGAAGTGTCGGAACGTTGC	sgRNA
**fdl_FP**	CGCGGACTTCTCCTTGACACAG	DNA oligo
**fdl_RP**	CGAACCCGCAGTCCAGGTAC	DNA oligo
**Dronc sg1**	CGTCGAGGACCTCAAGCATT	sgRNA
**Dronc sg2**	ACTATCAGGTAAGAGAAAGA	sgRNA[1]
**dronc_FP1**	ACCTCTGAGTTAGAAAGGGATCG	DNA oligo
**dronc_RP1**	ACCTACCTTTAGTGTCATCATCCA	DNA oligo
**dronc_FP2**	GAAAGAACACAGGGAAGCAATC	DNA oligo
**dronc_RP2**	CGCGATAAGTATGTCGGAGTAG	DNA oligo
**cDNA_FP1**	GCCAATGATCGAACCATTCAG	DNA oligo
**cDNA_RP1**	AATCTTTGTTGGCTCGAGAA	DNA oligo
**Actin FP1**	TCCCCATCTACGAAGGTTACGC	DNA oligo
**Actin RP1**	GCGGTGGTGGTGAAAGAGTAAC	DNA oligo
**pF-eGFP**	GAACCCATCGAGCTGAA	DNA oligo
**pR-eGFP**	TGCTTGTCGGCCATGATATAG	DNA oligo
**Probe-eGFP**	TTGCCGTCCTCCTTGAAGTCGAT	PrimeTime<br>5' 6-FAM/ZEN/3' IBFQ
**pF-ie-1**	TGCCACACTTGCAGCTC	DNA oligo
**pR-ie-1**	ATTTGCATACAACAAGTACACTGC	DNA oligo
**Probe-ie-1**	ATG TGT GCG CGT TAC CAC AAA TCC	PrimeTime<br>5' HEX/ZEN/3' IBFQ

* Sequence for Fdl sg taken from Mabashi-Asazuma and Jarvis (2017)
[1] sgRNA on minus strand

Table S2 PCR conditions for Platinum™ SuperFi II Green PCR Master Mix

Cycles	Temperature [°C]	Time
**1**	98	30 sec
**35**	98	10 sec
	60	10 sec
	72	15 sec/kb
**1**	72	5 min
**1**	4	∞
